# Sensory Drivers of Consumer Acceptance, Purchase Intent and Emotions toward Brewed Black Coffee

**DOI:** 10.3390/foods11020180

**Published:** 2022-01-11

**Authors:** Ammaraporn Pinsuwan, Suntaree Suwonsichon, Penkwan Chompreeda, Witoon Prinyawiwatkul

**Affiliations:** 1 Kasetsart University Sensory and Consumer Research Center (KUSCR), Department of Product Development, Faculty of Agro-Industry, Kasetsart University, Bangkok 10900, Thailand; ammaraporn.pi@gmail.com (A.P.); fagipkc@ku.ac.th (P.C.); 2 Agricultural Center, School of Nutrition and Food Sciences, Louisiana State University, Baton Rouge, LA 70803, USA; wprinya@lsu.edu

**Keywords:** coffee, emotions, descriptive analysis, consumer perception, liking

## Abstract

The link between coffee aroma/flavor and elicited emotions remains underexplored. This research identified key sensory characteristics of brewed black coffee that affected acceptance, purchase intent and emotions for Thai consumers. Eight Arabica coffee samples were evaluated by eight trained descriptive panelists for intensities of 26 sensory attributes and by 100 brewed black coffee users for acceptance, purchase intent and emotions. Results showed that the samples exhibited a wide range of sensory characteristics, and large differences were mainly described by the attributes *coffee identity (coffee ID)*, *roasted*, *bitter taste*, *balance/blended* and *fullness*. Differences also existed among the samples for overall liking, purchase intent and most emotion terms. Partial least square regression analysis revealed that liking, purchase intent and positive emotions, such as *active*, *alert*, *awake*, *energetic*, *enthusiastic*, *feel good*, *happy*, *jump start*, *impressed*, *pleased*, *refreshed* and *vigorous* were driven by *coffee ID*, *roasted*, *ashy*, *pipe tobacco*, *bitter taste*, *rubber*, *overall sweet*, *balanced/blended*, *fullness* and *longevity.* Contrarily, *sour aromatic*, *sour taste*, *fruity*, *woody*, *musty/earthy*, *musty/dusty and molasses* decreased liking, purchase intent and positive emotions, and stimulated negative emotions, such as *disappointed*, *grouchy* and *unfulfilled*. This information could be useful for creating or modifying the sensory profile of brewed black coffee to increase consumer acceptance.

## 1. Introduction

Coffee is one of the most widely consumed beverages in the world. In 2021, revenue in the coffee segment amounts to around $409 billion, and the global coffee market is anticipated to grow annually by 6.9% during 2021–2025 [1]. Coffee-drinking is driven by different motivations, such as the psychological stimulation induced by caffeine and the sensory enjoyment provided by coffee aroma and flavor [2]. Descriptive sensory analysis was used in several studies as an effective evaluation method to determine the effects of various factors (e.g., coffee origin, roast level, brewing method, serving temperature) on coffee aroma and flavor [3,4,5,6,7]. Chambers et al. [8] developed a sensory lexicon containing a set of 110 well-defined and referenced attributes, which could be used to describe the aroma and flavor of a wide range of brewed coffee samples.

Recently, the emotional experiences elicited by foods and beverages have increasingly gained interest from researchers, because they are known to affect consumer acceptance and consumption, apart from product sensory characteristics [9]. The EsSense Profile™ (ESP) with 39 emotion terms developed by King and Meiselman [10] has been widely used to measure emotional responses toward food products [11]. As different foods evoke different emotions [12], emotion scales were developed for specific food products, such as dark chocolate [13], chocolate and hazelnut spreads [14], blackcurrant squashes [15], wine [16], beer [17], ice cream [18], muffin [19], and entomophagy [20]. For coffee, an emotion lexicon called the Coffee-drinking Experience (CDE) was developed by Bhumiratana et al. [21]. The CDE consists of 44 emotion terms that describe coffee-drinking experiences in two dimensions, a valence dimension (unpleasant-pleasant) and an activation dimension (low-high). With the CDE, more specialized emotions that described *alert* and *focused* mental states (e.g., *in control*, *motivated*, *productive*, *clear minded*) were captured. Later, Kanjanakorn and Lee [22] used the ESP and CDE to study emotions elicited by coffee-drinking, and results of the two scales were compared. They found that emotions evoked by coffee-drinking were captured as *energetic*, *satisfied* and *pleased* by both scales at similar ratings. However, additional significant changes in *boosted*, *jolted* and *jump start* were detected when the CDE was used. van Zyl and Meiselman [23] suggested that a language-specific emotion lexicon was necessary because studies in different countries revealed that emotions expressed by words were influenced by culture and context [14,24,25]. Thus, coffee emotion lexicons were developed in different languages, such as Chinese, Korean [26] and Thai [27] using the CDE and/or the ESP as baselines. 

While emotions toward food products have been studied extensively, the link between emotion responses and product sensory characteristics remains underexplored. Research in this area is limited only to a few product categories, such as yogurt [28,29], chocolate/dark chocolate [13,30,31,32], cashew nut, processed tomato [31,32], potato chips [29,32], cheese, snack bars, and fruit [29]. Based on the results of previous research, the association between emotions and sensory characteristics was found to be specific to a product category, that is, the association found in one product category could not be extended to other product categories [11]. For example, *sweet* in cashew nut was found to be positively linked with *affectionate* and *aggressive*, and negatively with *bored*, while *sweet* in chocolate was found to positively link with *guilty*, and negatively with *interested* and *satisfied*. In canned tomato, *sweet* was associated with *curiosity*, *surprise* and *enthusiasm* [32]. To our knowledge, the work done by Bhumiratana et al. [33] was the only research that studied the link between emotions and sensory characteristics of brewed coffee using data obtained from consumer testing and sensory descriptive analysis. In their study, six coffee samples commercially available in the US market were evaluated by a trained coffee panel to rate the intensities of 12 sensory attributes. The same set of samples were used to elicit emotions in 94 US coffee drinkers. Results revealed key sensory characteristics of brewed coffee that stimulated positive and negative emotions for the US consumers. 

The current research was aimed to study further on the sensory characteristics of brewed coffee that drove acceptance, purchase intent and emotion responses for Thai consumers, another group of coffee users. Recently, coffee-drinking has become a new cultural practice among Thai people and the coffee market in Thailand is expected to grow at an annual rate of 12.35% during 2021–2025 [34]. Thailand is also one of the top coffee producers, ranked third among the coffee-producing countries in Asia, following Vietnam and Indonesia [35]. As black coffee-drinking is becoming more popular [36], all samples in this study were evaluated in the form of brewed black coffee without the addition of other ingredients, such as sugar, milk and coffee creamer, and all of the participants for consumer test were brewed black coffee users.

## 2. Materials and Methods

### 2.1. Coffee Samples

Eight commercial Arabica roasted coffee bean samples were used in this study (Table 1). The samples were selected through a two-step screening process. Firstly, 87 coffee bean samples commercially available in Thailand were reviewed by researchers, and 15 samples were chosen to represent a diversity in roast levels (light, medium and dark) and manufacturing countries (Thailand, Lao People’s Democratic Republic, Ireland, Italy and USA). Secondly, brewed black coffee drinks prepared from those 15 samples were tasted by eight trained panelists, and eight samples that represented a wide range of sensory characteristics were selected for the current study.

### 2.2. Sample Preparation

Each of the coffee samples was prepared by grinding the roasted beans to a medium grind using a coffee bean grinder (DēLonghi, model KG 89, Treviso, Italy); this was done no more than 15 min before brewing to preserve the volatile compounds and freshness of the coffee samples [5]. A drip coffee maker was used to brew the coffee samples in this study. According to Sanchez and Chambers [5], dripping was recommended as the most appropriate brewing method for use in sensory testing of coffee over cupping, espresso and filtered infusion methods because the dripping method provided better differentiation among coffee samples in terms of aroma, flavor and aftertaste attributes. Purified (reverse osmosis, carbon filtered) water (1000 mL) at room temperature was added to a drip coffee maker machine (Bosch, model TKA3A031, Munich, Germany). Ground coffee (55 g) was put into a coffee filter (1 × 4-inch, Bon Café, Bangkok, Thailand) and then into the coffee-maker machine. The ratio of ground coffee to water used in this study was in accordance with the International Standard for the preparation of coffee samples for use in sensory analysis [37], which suggested a ratio in a 5–9 g range per 100 mL of water, and was the same ratio that Di Donfrancesco et al. [4] used. Each brewed coffee sample was transferred into a stainless steel vacuum jug (Zebra, Bangkok, Thailand) to maintain its temperature until served. The serving temperature was in a range of 60–65 °C, which was similar to that used by Di Donfrancesco et al. [4].

### 2.3. Sample Evaluation 

All samples were subjected to a descriptive analysis and consumer test. All testing was conducted at the sensory evaluation facilities of the Department of Product Development at Kasetsart University, Bangkok, Thailand. The rooms had appropriate lighting, were air-conditioned (about 25 °C), and did not have extraneous odors. The testing room was separated from the sample preparation room. 

#### 2.3.1. Descriptive Analysis 

A panel consisting of eight trained panelists (all females, age range 38–56 years) from the Kasetsart University Sensory and Consumer Research (KUSCR) Center participated in the test. The panelists had 120 h of training in descriptive analysis and a minimum of 2000 h of testing experiences with various food products and beverages, including brewed coffee. The number of panelists used in this study fell within the range of 8–12, as suggested by Heymann et al. [38] for descriptive analysis. Previously, Maximo-Gacula and Rutenbeck [39] and Drake [40] indicated that a sensory panel with 6 to 14 trained panelists was adequate for descriptive analysis.

Prior to testing, 3 days of orientation (6 h each day) were held, during which panelists identified terms for describing flavor characteristics of all eight black coffee samples. To assist in identifying attributes, terminology developed by Sanchez and Chambers [5] and Chambers et al. [8] for brewed coffee was provided as an initial list. Panelists tasted all samples, discussed possible terms, and compiled a final consensus list of attributes for testing. Panelists were allowed to add new sensory terms detected in the samples which were not in the initial list. Subsequently, they discussed the definition, references and reference intensities of each attribute. During the orientation, panelists also practiced scoring of each attribute on a 15 cm line scale, with 0 meaning none and 15 meaning extremely high. Scores were monitored to ensure that variations among panelists were low (standard deviation ± 1) prior to product testing [41].

Product testing was completed in five 3 h sessions, with three to four samples being tested during each session. Two replicates were evaluated for each sample, and the evaluation was completed by replication. A randomized complete block design was used to determine the serving order within each replication. Panelists received 80 mL of each coffee sample in a pre-heated white porcelain cup labelled with a three-digit random code and covered with a lid. After tasting the sample, panelists rated the intensity of all attributes on 15 cm line scales (0 = none and 15 = extremely high) before moving onto the next sample. References were provided during evaluations to anchor values on the scale. Unsalted crackers (Jacob’s Original Cream Cracker, Kraft Foods Malaysia, Petaling Jaya, Malaysia), sliced apples and reverse osmosis deionized water were provided for panelists to cleanse their palates between samples. The panelists had at least a 15 min break after each sample evaluation.

#### 2.3.2. Consumer Test 

One hundred Thai consumers (62 females and 38 males, age range 18–64 years) participated in the test. They were recruited based on their weekly brewed black coffee consumption of at least three times a week. Of the 100 participants, 53 drank brewed black coffee 3–5 times a week, while 32 did once daily, and 15 did more than once daily. Bhumiratana et al. [21] classified participants who drank coffee 1–2 times a week, 3–5 times a week, and at least once daily as ‘light’, ‘medium’, and ‘heavy’ users, respectively. Thus, according to these authors, the consumer participants of this study consisted of 53 medium users and 47 heavy users of brewed black coffee.

Each consumer evaluated all eight black coffee samples in two sessions, four samples per session with a 5 min break between samples and 20 min break between sessions. Samples were served one at a time in a randomized and balanced order across consumers. After receiving 40 mL of each coffee sample in a paper cup, consumers were instructed to taste the sample and rate their overall liking on a 9-point hedonic scale (1 = dislike extremely, 9 = like extremely) and purchase intent on a 2-point category scale (purchase/not purchase). The consumers also checked all appropriate terms listed on a check-all that-apply (CATA) question to describe their emotions as evoked by each coffee sample. The CATA question included 20 emotion terms (Table 2) selected from our previous study [27], in which a consumer-defined emotion lexicon for coffee-drinking was developed and further refined by Thai coffee drinkers. Of the 20 terms, 15 terms were similar to those listed in the EsSense Profile^TM^ (ESP) [10], WellSense Profile^TM^ [42] and/or Coffee-drinking Experience (CDE) Profile [21], while five terms were newly generated by Thai coffee drinkers. Terms were presented in a random order among consumers in accordance with other studies [15,26] to minimize the visual processing effect on participants [43]. Unsalted crackers (Jacob’s Original Cream Cracker, Kraft Foods Malaysia, Petaling Jaya, Malaysia) and purified bottled water (Nestlé Pure Life^®^, Nestlé Thai, Ayuthaya, Thailand) were provided for consumers to cleanse their palates between samples.

### 2.4. Data Analysis

Multivariate analysis of variance (MANOVA) and Duncan’s multiple range test (DMRT) were performed to determine differences among eight brewed coffee samples based on attribute intensities, liking scores, and percentages of purchase intent at a 95% confidence level (*p* ≤ 0.05). Principal component analysis (PCA), with varimax rotation, was performed to uncover relationships between significant attributes and samples. Cochran’s Q test was used to determine significant differences among the coffee samples based on the frequency counts of each emotion term included in the CATA questions at a 95% confidence level (*p* ≤ 0.05) [44]. When significant differences were found, the Marascuilo and McSweeney procedure [44] was used for multiple pairwise comparisons. Correspondence analysis (CA) was then performed to determine relationships between significant emotion terms and samples. Partial least square regression (PLSR) analysis was also performed to determine sensory drivers of liking, purchase intent, and emotions using sensory attribute intensities as *x* variables and liking scores, purchase intent (%) and emotions (proportion of frequency counts) as *y* variables. Insignificant sensory attributes and emotion terms were excluded from the data set prior to PLSR analysis. The statistical software used for MANOVA and DMRT was IBM SPSS Statistics version 28.0 (Thaisoftup Co., Ltd., Bangkok, Thailand) and that used for PCA, Cochran’s Q test, CA and PLSR was XLSTAT statistical software version 19.6 (Addinsoft, New York, NY, USA). 

Although sample description was given (Table 1), sample identification was not revealed when reporting the results to avoid conflicts of interest. The three-digit random codes were used to denote the samples.

## 3. Results and Discussion

### 3.1. Sensory Characteristics 

Table 3 shows the final list of 26 attributes detected in the coffee samples along with their definitions, references and reference intensities for sensory evaluation. Most of the terms were consistent with those in the coffee lexicons developed by Chambers et al. [8] and Sanchez and Chambers [5] (*n* = 22 and 2, respectively), while two terms (*bitter aromatic* and *creosote/tar*) were newly added to the list. The definitions of the pre-existing attributes were consistent with those of previous studies; however, references for most of these attributes (*n* = 17) were modified because the ones used in the previous studies were not available in Thailand. Newly developed references were selected through a long discussion process among all panelists to fit with the attribute definitions [41]. Chambers et al. [8] introduced the concept of ‘living’ lexicons, that is, lexicons must be allowed to grow, change, or adapt as new samples are tested or new understandings of the attribute dimensions and new references arise. Thus, the current research expanded the pre-existing lexicons for brewed coffee by providing new sensory terms and references. A lexicon with well-defined and referenced descriptors helps to facilitate accurate and precise communication among the sensory panelists [45].

Mean intensities of sensory attributes for eight brewed black coffee samples are shown in Table 4. Most attributes were present in all samples, but at varying intensities. The attributes *fruity* and *rubber* were present only in a few samples (samples 562 and 187 for *fruity* and sample 712 for *rubber*), and thus described the unique sensory characteristics of the samples. MANOVA results revealed that almost all attributes were significantly different (*p* ≤ 0.05) among samples, except *nutty*. Large differences across the samples were mainly described by *coffee ID*, *roasted*, *bitter taste*, *balance/blended* and *fullness*.

**Figure 1:**
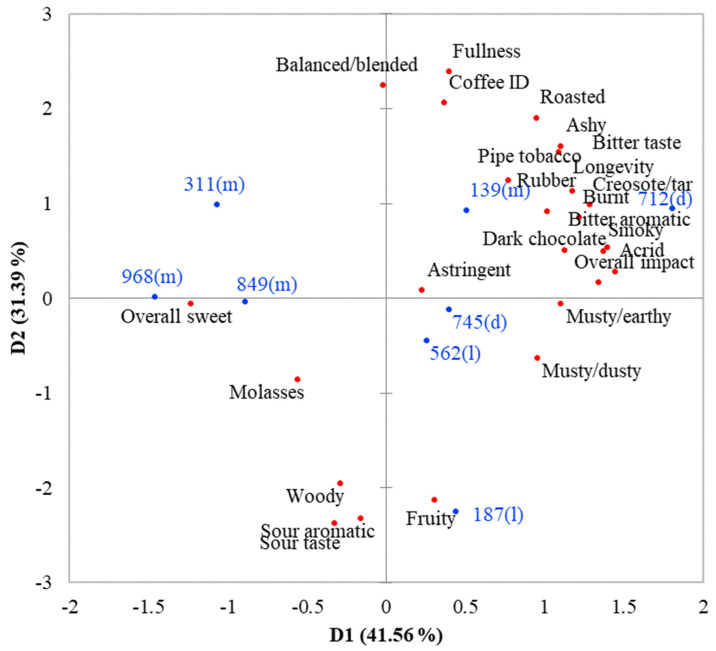
Principal component analysis (PCA) map of the sensory profiles of eight brewed black coffee.● = sensory attributes, ● = samples with roast level in parenthesis (l = light, m = medium and d = dark).

Results from the PCA map (Figure 1) indicated that significant sensory attributes formed two key dimensions that explained 72.9% of the total variability (41.6% and 31.3%, respectively). For principal component (PC) 1, heavily loaded attributes with absolute loading values greater than 0.6 included *burnt*, *acrid*, *bitter aromatic*, *smoky*, *ashy*, *musty/dusty*, *musty/earthy*, *creosote/tar*, *rubber*, *bitter taste*, *overall impact* and *longevity* in the positive dimension and *overall sweet* and *dark chocolate* in the negative dimension. For PC2, the heavily loaded attributes in the positive dimension were *coffee ID*, *roasted*, *balance/blended* and *fullness*, while those in the negative dimension were *woody*, *fruity*, *sour aromatic* and *sour taste*. Spreading of the samples over the PCA map indicated that the selected samples represented a wide range of sensory characteristics as intended. 

Sample 712, located on the far-right of the map (Figure 1), was differentiated from the rest of the samples by having the highest (*p* ≤ 0.05) intensities in *burnt, acrid, ashy, creosote/tar, bitter taste* and *overall impact* (Table 4). Additionally, it tended to be rated higher in *bitter aroma, smoky, pipe tobacco, musty/dusty, musty/earthy, longevity, coffee ID, roasted, balance/blended* and *fullness*, but lower in *overall sweet*, *woody*, *sour aromatic* and *sour taste* than other samples. Sample 712 was the only sample with a *rubber* note, even though such a flavor was detected at very low intensity. It was also the only sample with no detectable *molasses* flavor.

Samples 139, 562 and 745 shared some common characteristics, as shown by a closer grouping on the PCA map (Figure 1). These samples were rated as high as (*p* > 0.05) sample 712 in *coffee ID* (Table 4). In addition, their intensities in *burnt, bitter aromatic, smoky, ashy, creosote/tar* and *bitter taste* were second to those of sample 712 and were higher (*p* ≤ 0.05) than those of other samples. However, differences still existed among samples 139, 562 and 745. Sample 139 was different from the other two samples by having higher (*p* ≤ 0.05) *smoky, ashy, roasted* and *fullness* intensities, but lower (*p* ≤ 0.05) *woody, sour aromatic* and *sour taste* intensities. Sample 562 was rated higher (*p* ≤ 0.05) in *fruity, sour aromatic* and *sour taste,* but lower (*p* ≤ 0.05) in *bitter taste, dark chocolate* and *fullness* than the others, while sample 745 was higher (*p* ≤ 0.05) in *musty/dusty* and *dark chocolate*, but lower (*p* ≤ 0.05) in *pipe tobacco* and *astringent* than the others.

Samples 311, 849 and 968 were located closer to each other on the left of the PCA map (Figure 1) so they shared some common characteristics. These samples tended to be rated lower in *burnt, acrid, bitter aromatic, smoky, musty/dusty, musty/earthy, creosote/tar, bitter taste, overall impact* and *longevity* than the rest of the samples (Table 4). Differences among these three samples existed, especially between samples 311 and 968. Of the three samples, *woody* and *sour aromatic* were the lowest (*p* ≤ 0.05) for sample 311, while *coffee ID, roasted, bitter aromatic, ashy, molasses dark chocolate, musty/dusty, bitter taste* and *balance/blended* were the lowest (*p* ≤ 0.05) for sample 968. 

Sample 187, located at the bottom of the map (Figure 1), was differentiated from the rest of the samples by having the highest (*p* ≤ 0.05) intensities in *fruity, sour aromatic* and *sour taste,* but the lowest (*p* ≤ 0.05) intensities in *coffee ID, balance/blended* and *fullness* (Table 4). Additionally, it tended to be rated higher in *woody* and *must/dusty*, but lower in *roasted, burnt, ashy* and *bitter taste* than other samples.

Differences in sensory characteristics across the eight brewed black coffee samples were mainly due to differences in the roast level of the coffee beans. Generally, intensities of *coffee ID, roasted, burnt, acrid, bitter aromatic, smoky, ashy, dark chocolate, creosote/tar, bitter taste* and *fullness* tended to increase with an increased roast level from light to dark (Table 4). On the other hand, *sour aromatic* and *sour taste* intensities tended to decrease with the degree of roasting. The increases in *coffee ID, roasted, burnt, acrid, smoky* and *ashy* notes with an increased roast level have been reported by other researchers [3,33,46,47], while the decreases in *sour* characters with an increased roast level were in agreement with the studies of Bhumiratana et al. [33] and Akiyama et al. [47]. Roasting is one of the important factors that affects the sensory properties of coffee [48]. During the roasting process, coffee beans are exposed to a high temperature, and sugars in the beans undergo a caramelization reaction. Once the beans are heated to 205 °C, thermal decompositions and chemical changes occur, resulting in development and degradation of various volatile compounds, such as carbon dioxide, aldehydes, ketones, ethers, acetic acid, methanol, oils, and glycerol [3]. Thus, the temperature and condition during the roasting process are among the main factors that control the complexity of coffee aroma and flavor. An exception was observed for sample 562. Although it was labelled as a light roasted coffee, its *coffee ID, roasted* and *burnt* intensities were as high as those of other medium and dark roasted coffees. Apart from a roast level, other factors, such as the growing region/condition and processing methods from coffee cherries to green coffee beans [33] could have an impact on the aroma and flavor of coffee.

### 3.2. Consumer Liking, Purchase Intent and Emotions 

Sample 712 received the highest overall liking score; however, it was not significantly different (*p* > 0.05) from samples 139 and 311 (Table 5). These three samples were scored higher than six (like slightly) on a 9-point hedonic scale. On the contrary, sample 187 received the lowest (*p* ≤ 0.05) liking score that was in the range of ‘dislike slightly’ to ‘neither like nor dislike’, while scores of samples 849, 745, 968 and 562 were in the middle range of the scale (5.6–5.7) and were not significantly different (*p* > 0.05) from one another. The purchase intent also was significantly (*p* ≤ 0.05) different among samples and followed a similar trend of liking scores. 

Results indicated that liking scores and purchase intent tended to increase as roast levels increased from light to dark. However, an exception was observed for sample 745. Although the sample was a dark roasted coffee, it was rated lower in liking and purchase intent than other medium roasted coffee samples. Previously, Bhumiratana et al. [21,33] found that liking scores of one consumer cluster (*n* = 10) toward brewed coffee tended to decrease with increased roast levels (*n* = 10); however, no consistent trend was observed for other consumer clusters (*n* = 84) regarding the effect of roast levels. 

**Figure 2:**
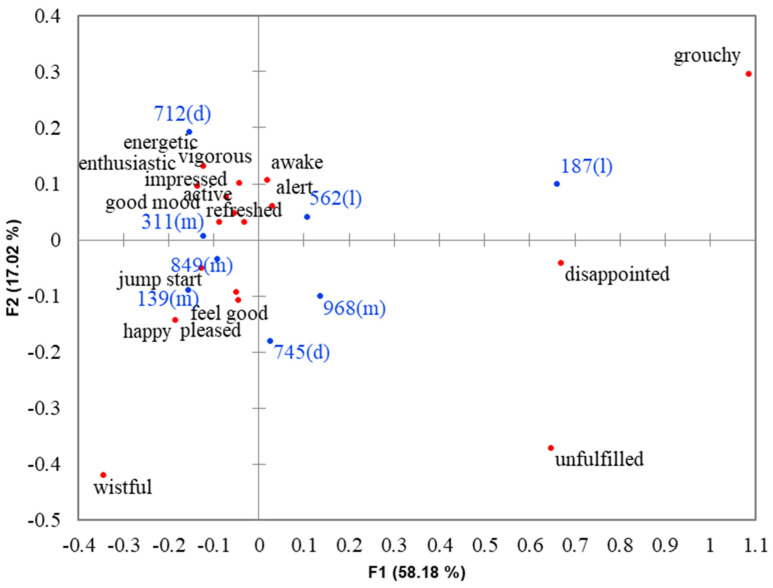
Correspondence analysis (CA) map showing the positioning of eight brewed black coffee samples in the emotion space. ● = emotion terms, ● = samples with roast level in parenthesis (l = light, m = medium and d = dark).

Results based on Cochran’s Q test revealed that differences existed across the eight coffee samples for 17 out of 20 emotion terms. The non-discriminating emotion terms were *bored, joyful* and *relaxed*. A symmetrical correspondence analysis (CA) map (Figure 2) shows the positioning of each coffee sample in the emotion space that explains 75.2% of total variability. The top three most liked samples (712, 139 and 311) were on the left quadrants and were explained by the emotion terms *active, alert, awake, energetic, enthusiastic, feel good, good mood, happy, impressed, jump start, pleased, refreshed, vigorous* and *wistful*. Next, on the right of these samples were samples 745, 968 and 562, which received liking scores in a neutral range. Although sample 849 was also rated in a neutral range for overall liking, its position in the CA map was closer to the top three most liked sample. By contrast, sample 187 that was disliked by consumers was on the right quadrant and was anchored by the terms *disappointed, grouchy* and *unfulfilled*. Results clearly showed that positive emotions were elicited by coffee samples that received liking scores in positive and neutral ranges of the scale, with generally higher frequency counts of positive emotions for liked samples than for neutrally liked samples, while negative emotions were evoked by disliked coffee samples. These results were supported by previous works on coffee [21,26,33] and other food categories, such as sweetener [49], salty snack, yogurt, cheese [29], beef [50] and fruit and vegetable juices [15,51] that samples with higher liking scores were more strongly associated with positive emotions, while less liked samples were more strongly associated with negative emotions. 

Product liking sometimes does not correlate well with emotions [52]. Even though liking scores of samples 712, 139 and 311 were not significantly different (*p* >0.05), sample 712 was described to stimulate positive, high-energy emotions such as *alert, awake* and *energetic,* more frequently (*p* ≤ 0.05) than the other two samples. Additionally, the feeling of *good mood* was mentioned more frequently (*p* ≤ 0.05) when drinking sample 849 compared to sample 745, although both samples received similar (*p* > 0.05) liking scores. Thus, the measurement of emotions elicited using a consumer-defined lexicon provided more discrimination across coffee samples than the hedonic measure, despite the fact that CATA emotion measures were only monitored in terms of presence/absence. The results concurred with those observed in the literature [15,21,32,53,54,55,56].

### 3.3. Sensory Drivers of Liking, Purchase Intent and Emotions

**Figure 3:**
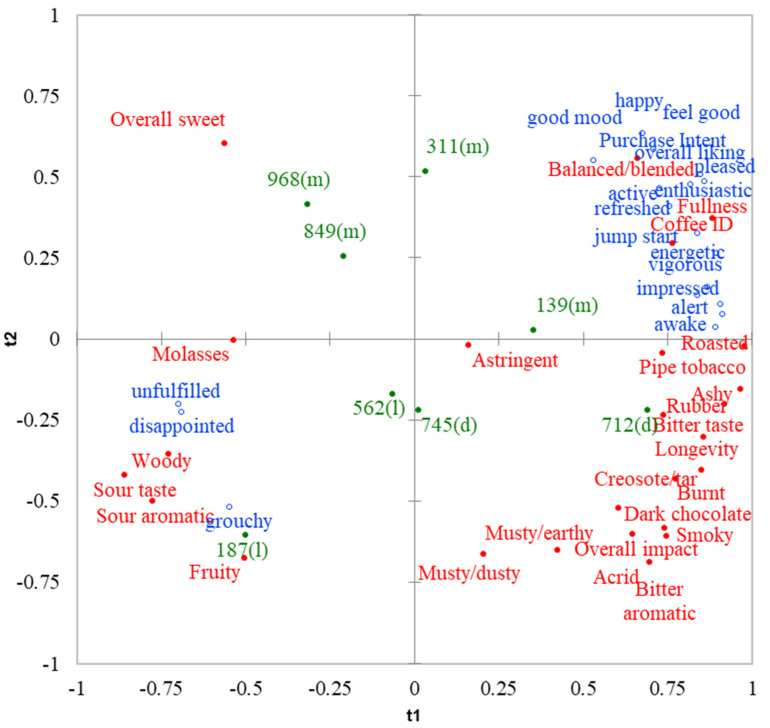
Partial least square regression map showing positioning of eight brewed black coffee samples in the sensory and emotion space. ● = sensory attributes, ο = overall liking/purchase intent/emotion terms, ● = samples with roast level in parenthesis (l = light, m = medium and d = dark).

Results from PLSR analysis (Figure 3) revealed sensory drivers of liking, purchase intent and emotions toward brewed black coffee based on 100 Thai coffee users. The first two components generated by PLSR explained 72.8% of total variability found in sensory attributes (*x*, explanatory variables) and 77.2% of total variability found in overall liking, purchase intent (%) and emotions (proportion of frequency counts) (*y*, dependent variables). Only the attributes with standardized β-coefficients in the PLSR models (data not shown) greater than 0.05 in absolute value were interpreted as the sensory drivers of these parameters [57]. It was found that liking, purchase intent and positive emotions including *active, alert, awake, energetic, enthusiastic, feel good, good mood, happy, jump start, impressed, pleased, refreshed* and *vigorous* were mainly driven by *coffee ID, roasted, balanced/blended* and *fullness. Ashy* and *pipe tobacco* also stimulated liking, purchase intent and almost all positive emotions except *good mood*. The *bitter taste* of brewed black coffee not only promoted liking, but also evoked an *impressed* feeling and positive high-energy feelings, including *alert, awake, energetic, jump start, refreshed* and *vigorous*. Unexpectedly, *rubber* was positively associated with liking, purchase intent and the feelings of *alert, awake, energetic, impressed, jump start, pleased, refreshed* and *vigorous*. This attribute was present in only one sample (sample 712) at very low intensity (Table 4). The consumers also related an *overall sweet* note to a *happy* feeling. *Longevity* of coffee flavor during tasting and after swallowing made the consumers feel *alert, awake* and *impressed*. 

On the contrary, *sour aromatic, sour taste, fruity, woody, musty/earthy, musty/dusty* and *molasses* negatively affected liking and purchase intent. In addition, the presence of these attributes generally stimulated negative emotions and/or suppressed positive emotions. Specifically, *sour aromatic, sour taste, fruity* and *woody* evoked *disappointed, grouchy* and *unfulfilled* feelings and suppressed all positive feelings. The presence of the *musty/earthy* flavor induced *grouchy,* and decreased *active, enthusiastic, feel good, good mood, pleased* and *happy* emotions. Similarly, a *musty/dusty* note elicited *grouchy* and decreased *feel good, good mood* and *happy* feelings. Although *molasses* did not evoke any negative emotions, it decreased the feelings of *pleased, feel good, refresh* and *jump start*. *Acrid* was also associated with the decrease of *happy* feeling. Therefore, these attributes were not desirable in the view of brewed black coffee users.

The positive impact *of Coffee ID* found in this study agreed with common knowledge that the coffee aroma/flavor generally induces positive feelings and is a key driver of coffee consumption [48,58]. Moreover, our findings on the positive effects of *roasted*, *pipe tobacco*, and *bitter taste* and the negative effects of *sour aromatic* and *sour taste* on emotions were consistent with those reported by Bhumiratana et al. [32] who determined sensory drivers for emotions toward brewed coffee using sensory descriptive data evaluated by trained panelists and emotion data evaluated by 94 US consumers. They found that *roasted* aroma and flavor aroused the feelings of *jump start, satisfied, boosted* and *special,* while *tobacco* flavor made the consumers feel *jolted* and *content*. In addition, *bitter taste* was reported to evoke *energetic* and *productive* feelings, while *acidity*, which corresponded to *sour aromatic* and *sour taste* in the current study, was found to arouse an *off-balance* feeling. However, a contradictory result was observed for the *coffee* aroma. Bhumiratana et al. [32] found the *coffee* aroma to associate with negative emotions (*bored, disgusted, annoyed* and *disappointed*) and attributed that to different understandings toward the term ‘*coffee*’ aroma in the views of consumers and trained panelists. Other attributes found to play an important role on consumer acceptance, purchase intent and emotions toward brewed black coffee in the current study (i.e., *balanced/blended, fullness, rubber, overall sweet,* *fruity, woody,* *musty/earthy, musty/dusty, molasses* and *acrid)* were not evaluated in the study of Bhumiratana et al. [32]. Another study by Hu and Lee [26] found that Korean and Chinese consumers did not like coffee with a strong *bitter taste*, which was contradictory to our findings. Disagreement between the two studies could be due to differences in terms of coffee sample context and consumer groups. In the current study, all samples being tested were black coffee, and all of the consumers participating in the test were black coffee users. Thereby, these consumers preferred coffee samples with a strong bitter taste, while in the study of Hu and Lee [26], various types of coffee, including the sweetened all-in-one type were evaluated, and participants were general coffee users.

It seems that most of the sensory attributes that drive acceptance, purchase intent and positive emotions while suppressing negative emotions for Thai consumers who drink brewed black coffee are the characteristics of dark roast coffees, which are the results of the roasting process. Product developers and coffee manufacturers could use the information obtained from this research in creating or modifying the sensory profile of coffee to increase consumer acceptance.

## 4. Conclusions

This research determined the sensory characteristics of brewed black coffee that affected consumer acceptance, purchase intent and emotions using data obtained from descriptive analysis and a consumer test. Results indicated that *coffee ID, roasted, ashy, pipe tobacco, bitter taste, rubber, overall sweet, balanced/blended, fullness* and *longevity* were the key sensory attributes driving liking, purchase intent and positive emotions, such as *active, alert, awake, energetic, enthusiastic, feel good, happy, jump start, impressed, pleased, refreshed* and *vigorous.* While *sour aromatic, sour taste, fruity, woody,* *musty/earthy, musty/dusty* and *molasses* decreased liking, purchase intent and positive emotions and stimulated negative emotions, such as *disappointed, grouchy* and *unfulfilled,* thus they were undesirable attributes. An increased roast level from light to dark tended to increase the intensities of desirable attributes (e.g., *coffee ID, roasted, ashy, bitter taste* and *fullness),* while decreasing the intensities of undesirable attributes (e.g., *sour aromatic* and *sour taste*). This information could be valuable for product developers and the coffee industry for creating or modifying the sensory profile of brewed black coffee to increase consumer acceptance. This research also expanded the pre-existing lexicons for brewed coffee by providing new sensory terms and references that could be used in sensory research.

## Figures and Tables

**Table 1:** A list of eight Arabica roasted coffee bean samples evaluated in the study.

Brand	Product Description	Manufacturing Country	Roast Level
Bon Café	Café Rama	Chonburi, Thailand	Light
Café Direct	Kilimanjaro	Dublin, Ireland	Light
Dao coffee	Arabica premium	Champasak, Lao People’s Democratic Republic	Medium
My Choice	Arabica blend	Samutsakhon, Thailand	Medium
Royal Project	Royal project coffee	Chiangmai, Thailand	Medium
The Coffee Bean Roasting	Roasted and whole bean coffee	Pathumthani, Thailand	Medium
Illy	Espresso	Trieste, Italy	Dark
Starbucks	Espresso roast	Seattle, WA, USA	Dark

**Table 2:** A list of 20 emotion terms with Thai translation evaluated in the study.

Emotion Terms (Thai Translation) *			
Active (กระฉับกระเฉง) ^a,b,c^	Energetic (มีแรง/มีพลัง) ^a,b,c^	Happy (มีความสุข) ^a,b^	Refreshed (สดชื่น) ^b^
Alert (ตื่นตัว) ^b^	Enthusiastic (กระตือรือร้น) ^a^	Impressed (ประทับใจ)	Relaxed (ผ่อนคลาย) ^b,c^
Awake (ตื่น) ^c^	Feel good (รู้สึกดี)	Joyful (สบายใจ) ^a,b,c^	Unfulfilled (ไม่เติมเต็ม) ^b^
Bored (เบื่อ/เซ็ง) ^a,c^	Good mood (อารมณ์ดี)	Jump start (พร้อมทำงาน) ^c^	Vigorous (กระปรี้ประเปร่า)
Disappointed (ผิดหวัง) ^c^	Grouchy (หงุดหงิด) ^c^	Pleased (พึงพอใจ) ^a,c^	Wistful (โหยหา)

* Emotion terms were selected from a previous study of Pinsuwan et al. [27] in which a consumer-defined emotion lexicon for coffee-drinking was developed and further refined by Thai coffee drinkers. Some of these terms were similar to those listed in other existing emotion profiles. ^a^ Terms that were also present in the EsSense Profile^TM^ (ESP) [10]. ^b^ Terms that were also present in the WellSense Profile^TM^ [42]. ^c^ Terms that were also present in the Coffee-drinking Experience (CDE) Profile [21].

**Table 3:** Sensory attributes, definitions and references for brewed black coffee evaluation.

Attribute	Definition	References and Intensities
Coffee identity (Coffee ID) ^a^	A distinctly roasted brown, slightly bitter aromatic characteristic of brewed coffee. Additional descriptors may/may not include woody, oily, acidic, and full bodied, and these notes may occur at varying intensities	Bon Aroma gold instant coffee ^1^ = 3.5 Giovanni Caffé American roasted & ground ^2^ = 7.0 Suzuki Coffee Arabica special blend ^2^ = 8.5
Roasted ^b^	Dark brown impression characteristic of products cooked to a high temperature by dry heat. It does not include bitter or burnt notes	Medium roasted peanuts ^3^ = 6.5 Dark roasted peanuts ^4^ = 9.5 Over roasted peanut ^5^ = 15.0
Burnt ^b^	The dark brown carbon impression of an over-cooked or over-roasted product that can be sharp, bitter and sour	Over roasted peanuts ^5^ = 7.5
Acrid ^c^	The sharp, pungent, bitter, acidic aromatics associated with products that are excessively roasted or browned	Dark roasted peanuts ^4^ = 3.0 Over roasted peanuts ^5^ = 8.5
Bitter aromatic ^d^	The perception of a bitter aromatic of coffee	Dark roasted peanuts ^4^ = 3.0 Over roasted peanuts ^5^ = 7.5
Smoky ^c^	An acute pungent aromatic that is a product of combustion of wood, leaves or non-natural product	Blue diamond smoked almonds = 2.0
Ashy ^c^	Dry, dusty, dirty or smoky aromatics associated with the residual of burnt products	Ash of Marlboro red cigarettes = 4.0 (only for smelling)
Woody ^c^	The sweet, brown, musty, dark aromatics associated with a bark of a tree	Heritage premium shelled walnuts = 4.0 Popsicle sticks = 7.5 (only for smelling)
Molasses ^b^	Dark caramelized top notes which may include slightly sharp, acrid and sulfur notes associated with molasses	Grandma’s molasses = 6.5
Overall sweet ^c^	The perception of a combination of sweet taste and aromatics	Post shredded Wheat = 1.5 Kellogg’s Special K Breakfast Cereal = 3.0
Nutty ^c^	A combination of slightly sweet, brown, woody, oily, musty, astringent, and bitter aromatics commonly associated with nuts, seeds, beans, and grains	Dr. Green wheat germ = 7.5
Dark chocolate ^c^	A high-intensity blend of cocoa and cocoa butter that may include dark roast, spicy, burnt, musty notes which include increased astringency and bitterness	Lindt Excellence dark chocolate bar (85% cocoa) = 11.0
Fruity ^c^	A sweet, floral aromatic blend of a variety of ripe fruits	Ceres Hanepoot white grape juice = 2.5
Sour aromatic ^b^	An aromatic associated with the impression of a sour product	Bush’s pinto beans = 2.0
Pipe tobacco ^c^	The brown, sweet, slightly pungent, fruity, floral, spicy aromatics associated with cured tobacco	Captain Black pipe tobacco (gold) = 8.5 (only for smelling)
Musty/dusty ^c^	The aromatics associated with dry closed air spaces such as attics and closets. May be dry, musty, papery, dry soil or grain	Dr. Green wheat germ = 3.5
Musty/earthy ^c^	Somewhat sweet, heavy aromatics associated with damp black soil	Fresh beetroot cube = 7.0
Creosote/tar ^d^	A pungent chemical aromatic associated with unrefined crude oil products	Tar = 12.0 (only for smelling)
Rubber ^c^	A dark heavy slightly sharp and pungent aromatic associated with rubber	Mahakit Rubber bands = 5.0 (only for smelling)
Bitter taste ^b^	The fundamental taste factor associated with a caffeine solution	0.5 g/L caffeine solution = 6.5 0.6 g/L caffeine solution = 8.5 0.7 g/L caffeine solution = 10.0 1.0 g/L caffeine solution = 12.0
Sour taste ^b^	The fundamental taste factor associated with a citric acid solution	0.15 g/L citric acid solution = 1.5 0.5 g/L citric acid solution = 3.0
Astringent ^e^	A drying, puckering or tingling sensation on the surface and/or edge of the tongue and mouth	0.5 g/L Alum Solution = 2.5
Overall impact ^c^	The maximum overall sensory impression during the whole tasting time	Suzuki Coffee Arabica special blend ^2^ = 7.5 Giovanni Caffé American roasted & ground ^2^ = 9.0 Bon Aroma gold instant coffee ^1^ = 12.0
Balance/ Blended ^c^	The melding of individual sensory notes such that the products present a unified overall sensory experience as opposed to spikes or individual notes	Bon Aroma gold instant coffee ^1^ = 3.0 Giovanni Caffé American roasted & ground ^2^ =6.0 Suzuki Coffee Arabica special blend ^2^ = 10.0
Longevity ^c^	The time that the full integrated sensory experience sustains itself in the mouth and after swallowing	Suzuki Coffee Arabica special blend ^2^ = 7.5 Giovanni Caffé American roasted & ground ^2^ = 9.0 Bon Aroma gold instant coffee ^1^ = 12.0
Fullness ^c^	The foundation of flavor notes that give substance to the product. The perception of robust flavor that is rounded with body	Bon Aroma gold instant coffee ^1^ = 5.0 Giovanni Caffé American roasted & ground ^2^ = 7.5 Suzuki Coffee Arabica special blend ^2^ = 10.0

^a^ Attribute from a study of Sanchez and Chambers [5] with modification of reference samples. ^b^ Attribute from a study of Chambers et al. [8]. ^c^ Attribute from a study of Chambers et al. [8] with modification of reference samples. ^d^ Newly added attribute in the current study. ^e^ Attribute from a study of Sanchez and Chambers [5]. ^1^ Prepared by dissolving instant coffee (30 g) in hot (74 °C) water (1420 mL). ^2^ Prepared by brewing ground coffee (30 g) with water (1420 mL) using a drip coffee maker machine. ^3,4,5^ Prepared by roasting raw peanut in an oven at 218 °C for 12, 17 and 22 min, respectively.

**Table 4:** Mean intensity scores of sensory attributes for eight black coffee samples.

Attributes	Coffee Samples (Roast Level *)								F-Values
	562 (l)	187 (l)	139 (m)	311 (m)	849 (m)	968 (m)	745 (d)	712 (d)	
Coffee ID	9.53 ± 0.31 ^a^	6.68 ± 0.42 ^d^	9.59 ± 0.04 ^a^	8.89 ± 0.09 ^b^	8.72 ± 0.22 ^b^	8.41 ± 0.22 ^c^	9.31 ± 0.05 ^a^	9.58 ± 0.42 ^a^	94.90
Roasted	9.91 ± 0.04 ^b^	8.72 ± 0.13 ^c^	10.69 ± 0.35 ^a^	9.74 ± 0.46 ^b^	9.56 ± 0.27 ^b^	9.09 ± 0.13 ^c^	9.99 ± 0.19 ^b^	11.06 ± 0.35 ^a^	24.62
Burnt	5.36 ± 0.02 ^b,c^	4.91 ± 0.31 ^d^	5.42 ± 0.29 ^b^	4.77 ± 0.11 ^d^	4.83 ± 0.17 ^d^	5.05 ± 0.07 ^c,d^	5.57 ± 0.13 ^b^	5.94 ± 0.09 ^a^	12.89
Acrid	4.99 ± 0.07 ^b,c^	5.02 ± 0.33 ^b,c^	5.28 ± 0.40 ^b^	4.50 ± 0.09 ^d,e^	4.72 ± 0.13 ^c,d^	4.23 ± 0.33 ^e^	5.34 ± 0.13 ^b^	5.76 ± 0.02 ^a^	12.38
Bitter aromatic	5.28 ± 0.27 ^b,c^	5.02 ± 0.20 ^c,d^	5.39 ± 0.34 ^b,c^	4.81 ± 0.09 ^d^	4.76 ± 0.07 ^d^	4.38 ± 0.09 ^e^	5.59 ± 0.04 ^a,b^	5.89 ± 0.19 ^a^	15.10
Smoke	3.56 ± 0.04 ^b^	3.62 ± 0.17 ^b^	3.94 ± 0.26 ^a^	3.34 ± 0.13 ^b,c^	3.14 ± 0.19 ^c^	3.16 ± 0.04 ^c^	3.53 ± 0.08 ^b^	4.06 ± 0.18 ^a^	9.31
Ashy	1.94 ± 0.14 ^c,d^	1.67 ± 0.26 ^d,e^	2.56 ± 0.09 ^b^	1.96 ± 0.03 ^c,d^	1.92 ± 0.06 ^c,d^	1.61 ± 0.10 ^e^	2.00 ± 0.09 ^c^	2.94 ± 0.00 ^a^	20.51
Woody	1.48 ± 0.20 ^a^	1.53 ± 0.31 ^a^	1.19 ± 0.18 ^b^	1.10 ± 0.05 ^b^	1.50 ± 0.00 ^a^	1.49 ± 0.12 ^a^	1.49 ± 0.24 ^a^	1.20 ± 0.07 ^b^	3.72
Molasses	1.16 ± 0.04 ^a^	0.99 ± 0.11 ^a^	0.97 ± 0.04 ^a^	1.01 ± 0.11 ^a^	1.14 ± 0.02 ^a^	0.53 ± 0.04 ^b^	1.21 ± 0.34 ^a^	0.00 ± 0.00 ^c^	27.42
Overall sweet	1.78 ± 0.17 ^a^	1.44 ± 0.18 ^b,c^	1.51 ± 0.05 ^b,c^	1.67 ± 0.06 ^a,b^	1.79 ± 0.13 ^a^	1.78 ± 0.19 ^a^	1.60 ± 0.04 ^a,b^	1.29 ± 0.02 ^c^	4.83
Nutty ^ns^	1.34 ± 0.17	1.08 ± 0.24	1.29 ± 0.04	1.22 ± 0.04	1.38 ± 0.09	1.35 ± 0.18	1.39 ± 0.11	1.24 ± 0.07	1.74
Dark chocolate	2.81 ± 0.08 ^c^	2.48 ± 0.47 ^d^	3.06 ± 0.27 ^b^	2.39 ± 0.11 ^d^	2.38 ± 0.35 ^d^	1.94 ± 0.09 ^e^	3.59 ± 0.04 ^a^	3.14 ± 0.06 ^b^	36.29
Fruity	0.79 ± 0.03 ^b^	1.05 ± 0.12 ^a^	0.00 ± 0.00 ^c^	0.00 ± 0.00 ^c^	0.00 ± 0.00 ^c^	0.00 ± 0.00 ^c^	0.00 ± 0.00 ^c^	0.00 ± 0.00 ^c^	294.30
Sour aromatic	2.78 ± 0.49 ^b^	3.00 ± 0.09 ^a^	1.66 ± 0.12 ^e^	1.43 ± 0.19 ^f^	2.17 ± 0.24 ^c,d^	2.26 ± 0.28 ^c^	2.04 ± 0.18 ^d^	1.43 ± 0.17 ^f^	79.43
Pipe tobacco	1.28 ± 0.22 ^a,b,c^	1.04 ± 0.15 ^c,d^	1.41 ± 0.13 ^a,b^	1.16 ± 0.05 ^b,c^	1.03 ± 0.01 ^c,d^	1.03 ± 0.22 ^c,d^	0.86 ± 0.55 ^d^	1.44 ± 0.09 ^a^	5.53
Musty/dusty	1.63 ± 0.00 ^c,d^	1.93 ± 0.16 ^a,b,c^	1.60 ± 0.21 ^d,f^	1.66 ± 0.35 ^b,c,d^	1.81 ± 0.09 ^a,b,c,d^	1.33 ± 0.29 ^f^	2.01 ± 0.02 ^a^	1.95 ± 0.07 ^a,b^	5.48
Musty/earthy	1.34 ± 0.04 ^a,b,c^	1.29 ± 0.09 ^a,b,c^	1.48 ± 0.12 ^a^	1.09 ± 0.04 ^c^	1.19 ± 0.18 ^b,c^	1.11 ± 0.19 ^c^	1.46 ± 0.11 ^a,b^	1.33 ± 0.03 ^a,b,c^	2.76
Creosote/tar	1.36 ± 0.06 ^b^	0.99 ± 0.07 ^d^	1.36 ± 0.07 ^b^	0.94 ± 0.04 ^d^	1.04 ± 0.15 ^c,d^	0.97 ± 0.04 ^d^	1.26 ± 0.06 ^b,c^	1.75 ± 0.32 ^a^	11.75
Rubber	0.00 ± 0.00 ^b^	0.00 ± 0.00 ^b^	0.00 ± 0.00 ^b^	0.00 ± 0.00 ^b^	0.00 ± 0.00 ^b^	0.00 ± 0.00 ^b^	0.00 ± 0.00 ^b^	1.33 ± 0.02 ^a^	470.97
Bitter taste	10.22 ± 0.04 ^c^	9.38 ± 0.18 ^e^	10.79 ± 0.03 ^b^	10.18 ± 0.16 ^c^	9.76 ± 0.13 ^d^	9.44 ± 0.27 ^e^	11.04 ± 0.50 ^b^	11.59 ± 0.10 ^a^	52.43
Sour taste	1.98 ± 0.29 ^b^	2.33 ± 0.34 ^a^	1.10 ± 0.02 ^e^	1.13 ± 0.06 ^e^	1.61 ± 0.30 ^c,d^	1.79 ± 0.11 ^b,c^	1.51 ± 0.24 ^d^	0.98 ± 0.15 ^e^	30.37
Astringent	1.47 ± 0.06 ^a^	1.35 ± 0.04 ^a^	1.39 ± 0.06 ^a^	1.31 ± 0.09 ^a,b^	1.39 ± 0.00 ^a^	1.34 ± 0.27 ^a^	1.16 ± 0.01 ^b^	1.41 ± 0.04 ^a^	2.54
Overall impact	10.38 ± 0.09 ^b^	10.34 ± 0.39 ^b^	10.19 ± 0.34 ^b,c^	9.93 ± 0.25 ^c,d^	9.80 ± 0.02 ^d^	9.68 ± 0.07 ^d^	9.95 ± 0.02 ^c,d^	11.09 ± 0.31 ^a^	15.48
Balance/Blended	9.00 ± 0.44 ^a,b^	5.50 ± 0.27 ^e^	8.77 ± 0.24 ^b,c^	9.28 ± 0.40 ^a^	8.50 ± 0.35 ^c^	8.03 ± 0.13 ^d^	8.66 ± 0.04 ^b,c^	9.06 ± 0.22 ^a,b^	68.23
Longevity	10.16 ± 0.04 ^b,c^	9.88 ± 0.44 ^c,d^	10.44 ± 0.01 ^a,b^	10.05 ± 0.11 ^c,d^	9.69 ± 0.19 ^d,e^	9.46 ± 0.06 ^e^	9.78 ± 0.04 ^c,d,e^	10.70 ± 0.19 ^a^	9.61
Fullness	8.70 ± 0.02 ^d^	7.71 ± 0.47 ^e^	9.75 ± 0.18 ^a^	9.24 ± 0.34 ^b,c^	8.73 ± 0.03 ^d^	8.64 ± 0.02 ^d^	9.06 ± 0.18 ^c^	9.55 ± 0.28 ^a,b^	33.54

Scores were the average values of two replications, where each replication was evaluated by eight panelists. * Roast level: l = light, m = medium and d = dark. ^a,b,c,d,e,f^ When the F-test was significant (the critical F value = 2.21 at α = 0.05, df = 7, 49, a two-tailed test, the mean intensities in the same row with different superscripts are significantly different (*p* ≤ 0.05), based on Duncan’s multiple range test. ^ns^ Mean intensities in the same row are not significantly different (*p* > 0.05).

**Table 5:** Overall liking score and purchase intent of eight black coffee samples, as evaluated by 100 consumers.

Coffee Samples (Roast Level *)	Overall Liking Scores	% Consumers Who Would Purchase the Sample
712 (d)	6.5 ± 1.6 ^a^	75 ^a^
139 (m)	6.2 ± 1.6 ^a^	63 ^a,b,c^
311 (m)	6.1 ± 1.6 ^a^	69 ^a,b^
849 (m)	5.7 ± 1.7 ^b^	53 ^b,c^
745 (d)	5.7 ± 1.7 ^b^	53 ^b,c^
968 (m)	5.6 ± 1.7 ^b^	51 ^b,c^
562 (l)	5.6 ± 1.8 ^b^	48 ^c^
187 (l)	4.4 ± 1.9 ^c^	26 ^d^

Liking scores were the average values of 100 consumers. * roast level: l = light, m = medium and d = dark. ^a,b,c,d^ Values within columns with different superscripts are significantly different (*p* ≤ 0.05).

## Data Availability

The data presented in this study are available on request from the corresponding author.
